# A Case Series of Familial *ARID1B* Variants Illustrating Variable Expression and Suggestions to Update the ACMG Criteria

**DOI:** 10.3390/genes12081275

**Published:** 2021-08-20

**Authors:** Pleuntje J. van der Sluijs, Mariëlle Alders, Alexander J. M. Dingemans, Kareesma Parbhoo, Bregje W. van Bon, Jennifer C. Dempsey, Dan Doherty, Johan T. den Dunnen, Erica H. Gerkes, Ilana M. Milller, Stephanie Moortgat, Debra S. Regier, Claudia A. L. Ruivenkamp, Betsy Schmalz, Thomas Smol, Kyra E. Stuurman, Catherine Vincent-Delorme, Bert B. A. de Vries, Bekim Sadikovic, Scott E. Hickey, Jill A. Rosenfeld, Isabelle Maystadt, Gijs W. E. Santen

**Affiliations:** 1 Department of Clinical Genetics, Leiden University Medical Center, 2333 ZA Leiden, The Netherlands; p.j.van_der_sluijs@lumc.nl (P.J.v.d.S.); C.Ruivenkamp@lumc.nl (C.A.L.R.); 2 Department of Clinical Genetics, Amsterdam UMC, University of Amsterdam, 1105 AZ Amsterdam, The Netherlands; m.alders@amsterdamumc.nl; 3 Department of Human Genetics, Donders Institute for Brain, Cognition and Behaviour, Radboud University Medical Center, 6525 GA Nijmegen, The Netherlands; A.Dingemans@radboudumc.nl (A.J.M.D.); bert.devries@radboudumc.nl (B.B.A.d.V.); 4 Division of Genetic & Genomic Medicine, Nationwide Children’s Hospital, Columbus, OH 43205, USA; Kareesma.Parbhoo@nationwidechildrens.org (K.P.); Betsy.Schmalz@nationwidechildrens.org (B.S.); scott.hickey@nationwidechildrens.org (S.E.H.); 5 Department of Human Genetics, Radboud University Medical Center, 6525 GA Nijmegen, The Netherlands; Bregje.vanBon@radboudumc.nl; 6 Department of Pediatrics, University of Washington, Seattle, WA 98195, USA; joubert@uw.edu (J.C.D.); ddoher@uw.edu (D.D.); 7 Center for Integrative Brain Research, Seattle Children’s Research Institute, Seattle, WA 98101, USA; 8 Human Genetics and Clinical Genetics, Leiden University Medical Centre, 2333 ZA Leiden, The Netherlands; ddunnen@humgen.nl; 9 Department of Genetics, University of Groningen, University Medical Center Groningen, 9700 RB Groningen, The Netherlands; e.h.gerkes@umcg.nl; 10 Rare Disease Institute, Children’s National Hospital, Washington, DC 20010, USA; imiller@childrensnational.org (I.M.M.); dregier@childrensnational.org (D.S.R.); 11 Centre de Génétique Humaine, Institut de Pathologie et de Génétique, 6041 Gosselies, Belgium; stephanie.moortgat@ipg.be (S.M.); isabelle.maystadt@ipg.be (I.M.); 12 EA7364 RADEME, Institut de Génétique Médicale, Université de Lille, CHU de Lille, F-59000 Lille, France; Thomas.SMOL@chru-lille.fr; 13 Erasmus MC, Department of Clinical Genetics, University Medical Center Rotterdam, 3015 GD Rotterdam, The Netherlands; k.stuurman@erasmusmc.nl; 14 EA7364 RADEME, Université de Lille, Clinique de Génétique, CHU de Lille, F-59000 Lille, France; Catherine.DELORME@CHRU-LILLE.FR; 15 Verspeeten Clinical Genome Centre and London Health Sciences Centre, Department of Pathology and Laboratory Medicine, Western University, London, ON N6A 3K7, Canada; bekim.sadikovic@lhsc.on.ca; 16 Department of Pediatrics, The Ohio State University College of Medicine, Columbus, OH 43210, USA; 17 Department of Molecular and Human Genetics, Baylor College of Medicine, Houston, TX 77030, USA; jill.mokry@bcm.edu; 18 Baylor Genetics Laboratories, Houston, TX 77021, USA

**Keywords:** *ARID1B*, Coffin–Siris syndrome, ACMG guidelines, inherited, familial, variable expression, non-pathogenic, intellectual disability

## Abstract

*ARID1B* is one of the most frequently mutated genes in intellectual disability (~1%). Most variants are readily classified, since they are de novo and are predicted to lead to loss of function, and therefore classified as pathogenic according to the American College of Medical Genetics and Genomics (ACMG) guidelines for the interpretation of sequence variants. However, familial loss-of-function variants can also occur and can be challenging to interpret. Such variants may be pathogenic with variable expression, causing only a mild phenotype in a parent. Alternatively, since some regions of the *ARID1B* gene seem to be lacking pathogenic variants, loss-of-function variants in those regions may not lead to *ARID1B* haploinsufficiency and may therefore be benign. We describe 12 families with potential loss-of-function variants, which were either familial or with unknown inheritance and were in regions where pathogenic variants have not been described or are otherwise challenging to interpret. We performed detailed clinical and DNA methylation studies, which allowed us to confidently classify most variants. In five families we observed transmission of pathogenic variants, confirming their highly variable expression. Our findings provide further evidence for an alternative translational start site and we suggest updates for the ACMG guidelines for the interpretation of sequence variants to incorporate DNA methylation studies and facial analyses.

## 1. Introduction

Pathogenic variants in *ARID1B* are one of the top hits (~1%) in large-scale sequencing studies in intellectual disability (ID) populations [1,2] and *ARID1B* is also the most frequently mutated gene in Coffin–Siris syndrome (CSS) (OMIM 135900) (51–76%) [3,4,5]. With the increasing application of genome-wide screening approaches, such as exome and genome sequencing, more and more variants in *ARID1B* are identified. Usually variants in *ARID1B* can be readily classified, since most identified variants are de novo and predicted to lead to loss of function. According to the ACMG guidelines [6], this results in one very strong (PVS1) and one strong criterium (PS2), leading to a clinical classification as a pathogenic variant.

*ARID1B* variants are regularly identified in patients without specific features [7], complicating the interpretation of variants of uncertain significance (VUS). Indeed, we have previously reported on the variable expression of the *ARID1B* phenotype by showing IQ values within the normal range [7,8], suggesting that a variant classified as pathogenic could be inherited from a mildly affected parent. To make matters more complex, we also noticed that no pathogenic variants have been reported in the 5′ end of the 1542 bp exon 1 nor in the small 39 bp in-frame exon 3 of transcript NM_020732.3 [7], suggesting that loss-of-function variants in these regions may not be disease causing. Thus, interpretation of some loss-of-function variants, in particular those that are not proven de novo, may be challenging because of the difficulty to distinguish between variants not leading to disease and familial variants with variable expression.

To illustrate the difficulties in interpreting inherited loss-of-function variants in *ARID1B*, we describe here 12 index cases with potential loss-of-function variants that were either inherited or have an unknown inheritance. Furthermore, we describe our diagnostic evaluation to determine their true effect on clinical outcomes. This evaluation includes algorithms that combine regular facial recognition techniques with the modelling of human facial dysmorphisms [9], and DNA methylation patterns [10]. Together, this allowed for determining variants with variable expression versus non-pathogenic variants. Based on this cohort, we recommend updates for the ACMG guidelines for interpretation of sequence variants.

## 2. Materials and Methods

### 2.1. Patient Ascertainment

Patients with potential loss-of-function variants that were either inherited or of unknown inheritance were included. We included patients from colleagues who had approached us about such variants, screened variants using ClinVar, and identified additional patients through contacts with the Baylor Genetics Laboratories.

The institutional review board (IRB) of the Leiden University Medical Center, Leiden, The Netherlands, provided an approval waiver for this study.

### 2.2. Clinical Data Collection

Clinical information was collected through an online questionnaire or by asking the clinician to give a brief summary of their patient’s phenotype.

### 2.3. Transcript

Clinicians reported the genetic variant on transcript NM_020732.3, which is also used throughout the manuscript. The Matched Annotation from NCBI and EMBL-EBI (MANE) project (https://www.ncbi.nlm.nih.gov/refseq/MANE/ (accessed on 20 July 2021)), which aims to select a well-supported transcript for all genes to facilitate communication about variants, recently selected the NM_001374828.1 transcript for *ARID1B*. This transcript differs from the NM_020732.3 transcript, which is most widely used in clinical literature thus far, in three aspects: (1) the encoded protein has an N-terminal extension since an upstream start codon in exon 1 is used; (2) exon 3 of NM_020732.3 is not included; and (3) it has an additional in-frame 159 bp exon 11. The MANE project selected this transcript based on the conservation of the coding region and support for the transcription start site from FANTOM5 CAGE data as well as direct evidence of translation from uniquely mapping peptides (Human Peptide Atlas build 502). The MANE project also assigns the longest conserved coding region supported by evidence for the MANE Select. Where relevant, we mention the consequences of this transcript throughout the manuscript.

### 2.4. DNA Methylation

Analysis of the DNA methylation array data was performed by the clinical bioinformatics laboratory using Illumina Infinium EPIC arrays as previously described [11,12]. Methylation data for each sample were compared to the established DNA methylation signatures for *ARID1B* among 43 other disorders that were part of the EpiSign V2 clinical test. EpiSign analysis utilized the EpiSign Knowledge Database EKD, a clinical database with >5000 peripheral blood DNA methylation profiles, including disorder-specific reference cohorts and normal (general population samples with various age and racial backgrounds) controls housed at the London Health Sciences Centre Molecular Diagnostics Laboratory (https://www.lhsc.on.ca/palm/molecular.html (accessed on 15 January 2020)). Individual DNA methylation data for each subject were compared to the EKD using the Support Vector Machine (SVM)-based classification algorithm for EpiSign disorders. A Methylation Variant Pathogenicity (MVP) score was generated, ranging between 0 and 1, representing the confidence of the prediction for the specific class the SVM was trained to detect. Classification for a specific EpiSign disorder includes an MVP score assessment with a general threshold of >0.5 for positive, < 0.1 for negative and 0.1–0.5 for inconclusive or low-confidence samples; hierarchical clustering; and the multidimensional scaling (MDS) of a subject’s methylation data relative to the disorder-specific EpiSign probe sets and controls. A detailed description of this analytics protocol was described previously [11,13].

### 2.5. Analyses of Facial Features

#### 2.5.1. Computer Vision Algorithms

Using previously reported computer vision algorithms [9,14], we first assessed whether the photographs of the *ARID1B* patients with a confirmed pathogenic variant and who were referred to our national CSS expertise center in Leiden clustered compared to age-, ethnicity-, sex-matched controls with an ID. We performed this analysis with different age-groups (0–10 years, 10–18 years and 18+ years) as well, since we have observed in our CSS population that with advancing age, facial features may become less typical. We next investigated how photographs from patients with inherited *ARID1B* variants clustered. If more facial photographs of one patient were available, the photograph with the best quality for the analysis was chosen for the initial assessment.

#### 2.5.2. Face2Gene

We also analysed the photographs of our *ARID1B* familial cases by uploading them in Face2Gene (FDNA Inc., Boston, MA, USA). We assessed the rank of CSS in the top 30 suggested syndromes and their similarity to CSS according to Face2Gene on a scale of 0–1. Face2Gene analyses were performed in April–May 2021.

## 3. Results

**Figure 1:**
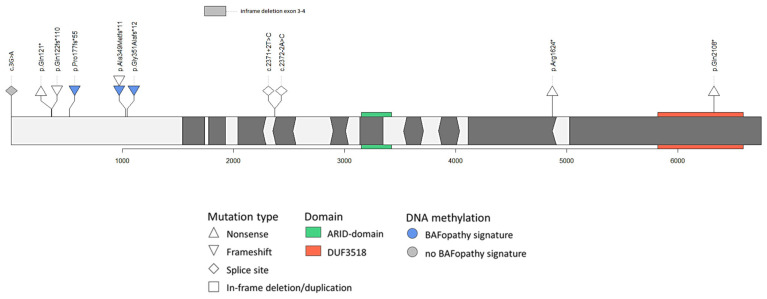
*ARID1B* variants, DNA methylation results and facial analyses of the included cases. Variants of the included *ARID1B* cases with their DNA methylation results (transcript NM_020732.3).

We collected 12 previously unreported index cases associated with a potential loss of function, as an inherited variant in *ARID1B* or a variant with unknown inheritance (Table 1 and Figure 1). Eight families had an inherited variant, one family consisted of two siblings of whom the mother was tested negative for the variant and there were three index cases with variants of unknown inheritance. The carrier parent of Case 11 is inconclusively mosaic (see also case description) and the parent of Case 12 is mosaic; the carrier parents of all other patients with inherited variants appeared to have non-mosaic or germline variants. In the DNA from peripherical blood, there was no indication for mosaicism in the other carrier parents.

### 3.1. DNA Methylation

*ARID1B* DNA methylation EpiSign analysis was performed for 5/12 cases where DNA and parental consent were available. A typical BAFopathy methylation pattern was detected in three familial cases (Table 1).

### 3.2. Facial Analyses

#### 3.2.1. Computer Vision Algorithms

Compared to matched controls, the photographs of patients with a confirmed pathogenic *ARID1B* variant (*n* = 34) did not cluster (*p* = 0.34). Selecting only *ARID1B* patients aged 10 and below (*n* = 21, Figure S1) showed that the photographs of this group did cluster (*p* = 0.038), while photographs of patients aged 10–18 and 18+ years did not cluster (respectively, *n* = 9, *p* = 0.73, and *n* = 4, *p* = 0.88).

The photographs of Index 1, 4, 5, 6, 7 and 12 and the parent of Index 12 clustered with photographs of the young *ARID1B* patients from our outpatient clinic compared to the controls, while the photographs of the parent of Index 2, 4 and 7 did not cluster (Table 1, Table S1 and Figure S1). The photographs of Case 2 at an age of two years and 11 years clustered with the young *ARID1B* patients, but her most recent photograph taken at an age of 11.5 years did not cluster.

#### 3.2.2. Face2Gene

Face2Gene suggested CSS as a possible syndrome for all pictures of children and of the parents of whom childhood pictures were available for analyses. CSS was the first suggested syndrome for the Cases 4, 6 and 7, and for childhood pictures of Parents 7 and 12 (Table 1 and Figure S2a,b). CSS was not suggested for the adult pictures of parents of Cases 2, 4 and 7. For Cases 2, 7 and 12, multiple facial photographs taken at different ages were available. The concordance of these photographs to the CSS Face2Gene model appears to decline with age (Figure S2c,d).

### 3.3. Variant Interpretation

#### 3.3.1. Variants at the 5′ end of Exon 1 of *ARID1B*

##### Case 1

This 20-year-old boy has a start codon variant, which was not found in his mother. It should be noted that on the MANE transcript NM_001374828.1, this variant is a missense variants (p.(Met84Ile)). He has moderate ID, normal speech development, seizures since the age of three years, cannot read or write and has small nails. According to the ACMG guidelines, this variant is classified as likely pathogenic (Table 1). The location of this variant and the absence of other pathogenic variants in the region make interpretation complex. His photograph clusters with other *ARID1B* patients, but the methylation pattern in his blood was not compatible with a BAFopathy. Given the lack of variants in the 5′ end of exon 1, our expert opinion is that the variant should be classified as a VUS.

##### Case 2

This six-year-old girl carries a paternally inherited nonsense variant in the 5′ end of exon 1 (c.361C>T, p.(Gln121*)). Her primary symptoms were dysmorphic features, hyperactivity and disruptive/defiant behaviour. None of these features were seen in the father and both father and daughter have normal intelligence. Although according to ACMG guidelines (Table 1) this variant would classify as likely pathogenic, taking our experience with the inheritance, location and phenotype into account, we would consider this variant a VUS. The photograph of the child at age two years clustered with other *ARID1B* patients; one of her photographs taken at 11 years also clustered, while another photo at this age did not cluster. The photograph of the parent at age 47 years did not cluster. No DNA samples were available for further testing. After adding the photograph data to our assessment, we still consider this variant a VUS.

##### Case 3

This is a singleton case aged >50 years from the AURORA study (conducted in the early 2000s in patients with end-stage kidney failure). A frameshift variant in the 5′ end of exon 1 was identified (c.363_364insG, p.(Gln122fs*110)). This variant has been reported several times in gnomAD. No additional information about this patient’s phenotype is available and inheritance was not tested. According to the ACMG guidelines (Table 1), this variant classifies as a VUS. However, based on the frequency in gnomAD and the reason why sequencing was performed, we consider this variant as likely benign. No photographs or DNA were available for further testing.

##### Case 4

This is a 17-month-old child who inherited a frameshift variant located at the 5′ end of exon 1 (c.521dup, p.(Pro177fs*55)) from a parent. Although based on ACMG guidelines this variant would be classified as likely pathogenic (Table 1), we initially considered this variant a VUS, based on the inheritance and the location at the 5′end of exon 1. The photograph of the child clustered with other *ARID1B* patients, while the photograph of the parent at age 36 years did not cluster. A picture of the parent during childhood was not available. DNA methylation in both the child and the parent was compatible with a BAFopathy. The phenotype of the child and the parent fit within the *ARID1B* spectrum and this variant is considered pathogenic.

##### Case 5

This is a 10-year-old boy who inherited a frameshift variant in exon 1 (c.1029_1056del, p.(Ala349Metfs*11)) from his mother. He has mild ID, speech delay, behavioural issues and attends special education school. His mother also attended special education school (as a child and adult) and has no behaviour issues. The maternal grandmother was also reported to have ID. The boy lives with his father. According to the ACMG guidelines this variant could be classified as likely pathogenic (Table 1). The photograph of this patient clustered with *ARID1B* patients, his DNA methylation pattern was compatible with a BAFopathy and this variant was considered pathogenic.

##### Case 6

This is a four-year-old girl with a maternally inherited frameshift variant in exon 1 (c.1044_1071del, p.(Ala349Metfs*11)). Further segregation analyses showed that the variant was present in another affected sibling and absent in an unaffected sibling. She has global developmental delay, severe speech delay, autistic features and feeding difficulties. Her mother has ID and her affected brother has developmental delay, mild ID and short stature. ACMG guidelines support a variant classification of likely pathogenic (Table 1). The photograph of the child clustered with *ARID1B* patients and the variant is considered pathogenic.

##### Case 7

This is a nine-year-old girl who inherited a frameshift variant in exon 1 (c.1044_1062del, p.(Gly351Alafs*12)) from her father. She had neonatal hypotonia with transient feeding difficulties, developmental delay (walked independently at 30 months, speech delay), mild ID (total IQ 73 at 7.5 years, verbal comprehension 84, perceptual reasoning 75, working memory 82, processing speed 76), behavioural problems (i.e. tantrums, ADHD), epilepsy and progressive obesity. The father had mild learning difficulties but no intellectual deficiency (total IQ 98 but heterogeneous profile, verbal comprehension 84, perceptual reasoning 106, working memory 112, processing speed 94). At physical examination, he had coarse facial features. He had epilepsy between 2 and 12 years of age. Based on the ACMG guidelines, this variant could be classified as likely pathogenic (Table 1). The photograph of the child clustered with *ARID1B* patients, while the photograph of her father at age 14 years did not cluster. The DNA methylation pattern in the parent and child was compatible with a BAFopathy. This variant is therefore classified as pathogenic.

#### 3.3.2. Splice Sites of in-Frame Exons or in-Frame Deletions in *ARID1B*

##### Case 8

This is a seven-year-old boy who inherited an in-frame deletion of exon 3 and 4 from his father. He has a clinical diagnosis of familial Mediterranean fever. A microarray was performed in search of an intragenic MEFV deletion because only one pathogenic variant in the FMF gene was identified. He has normal development and no dysmorphic features. ACMG guidelines support a classification as a VUS (Table 1). No photograph for facial analysis was available and DNA methylation did not show a BAFopathy pattern. This variant is considered likely benign.

##### Case 9

This is an 18-year-old girl with a splice site variant near the in-frame exon 7 (c.2371+2T>C) with unknown inheritance. This variant is located close to a variant (c.2371+5G>A, de novo) reported by Lord et al. [15]. She has severe ID, currently no speech, lost 3–4 words between age 1–2 years, seizures, myopia, hypoplasia of the corpus callosum, intracerebral lipoma of the quadrigeminal plate, self-injurious behaviour, mild scoliosis and camptodactyly of the 3rd and 5th finger. She is currently a senior in a high school program for individuals with developmental disabilities. Suggestive features for an *ARID1B*-related disorder in this case are hypoplasia of the corpus callosum, myopia, seizures and scoliosis. In accordance with the ACMG guidelines, this variant was considered a VUS (Table 1). No photographs or DNA were available for further testing.

##### Case 10

This is an 11-year-old girl who inherited a splice site variant near the in-frame exon 8 (c.2372-2A>C) from her mother. This variant has been reported in two siblings with autism spectrum disorder without ID who inherited this variant from their father. This variant was absent in their sibling without autism [16]. This variant was also reported in a stillborn case with features consistent with prenatal CSS with unknown inheritance [17].

She has severe global developmental delay, complex partial epilepsy, microcephaly, generalized hypotonia, cortical visual impairment, laryngomalacia, oromotor dysphagia, constipation, feeding difficulties and hip dislocation. No information about her mother is available. Although the combination of laryngomalacia with developmental delay, visual impairment and seizures is suggestive of *ARID1B*-related ID. The fact that the variant is inherited both in the literature case and in this family argues against pathogenicity. Unfortunately, no information on the mother, and no photographs or DNA could be obtained, and this variant remains a VUS, conforming to the ACMG guidelines (Table 1).

#### 3.3.3. Mosaicism in Parents

##### Case 11

The proband is an eight-year-old boy who has a sister with a nonsense variant in exon 18 (c.4870C>T, p.(Arg1624*)) that has been reported as pathogenic several times in the literature [7,18,19,20]. The mother tested negative for the variant and the father was potentially mosaic, with a very low signal on Sanger sequencing (data not shown). The proband has delayed motor milestones, delayed speech, moderate ID, autism spectrum disorder, hypotonia, dysmorphia, exotropia, short stature, hypoplastic left heart syndrome with coarctation of the aorta, undescended testes and inguinal hernias, feeding difficulty (G-tube dependent) and a history of strabismus. Brain MRI showed mild cerebellar vermis hypoplasia. His 10-year-old sister (diagnosed at 4.5 years) has developmental delay, moderate ID, no speech, behavioural anomalies, normal growth, no cardiac defect, feeding difficulties in infancy and, besides optic nerve hypoplasia, no brain anomalies. In accordance with the ACMG guidelines and based on the phenotype and previously reported loss-of-function variants located in this exon, this variant was considered pathogenic in the initial assessment (Table 1). No photographs could be obtained.

##### Case 12

This is a three-year-old boy (now eight years) who inherited a nonsense variant in exon 20 (c.6322C>T, p.(Gln2108*)) from his mosaic father (data not shown). Several patients with loss-of-function variants near this variant have been reported [3,4,5,7,19,20]. This boy’s phenotype matches the *ARID1B* phenotype well. His father is considered to have normal intellectual functioning, went to regular primary school and was able to keep up with peers, does not have developmental delay, does have a range of behavioural issues, mild scoliosis and currently lives independently with his partner and children. In accordance with the ACMG guidelines (Table 1), this variant was considered pathogenic. The photographs of the child at the age of eight years and the parent at the age of 18 years clustered with *ARID1B* patients, confirming the pathogenicity of this variant.

## 4. Discussion

### 4.1. Variable Expression

Variable expression of the *ARID1B* phenotype is evident in the inherited cases we have described (Table 1). For example, the parents of Cases 4–7 appear to be considerately less severely affected than their offspring. Although sporadic cases of inherited variants from similarly affected parents [21,22,23] or variants inherited through (gonadal) mosaicism [7,24,25,26,27] have been reported (Table S2), here we describe for the first time that a pathogenic, non-mosaic variant is inherited from a very mildly affected parent with normal IQ values (i.e., the father in Case 7). Unfortunately, the grandparents could not be tested in our cases with inherited variants. Parents in our cases were generally less severely affected than their child. This is likely explained by selection bias since mildly affected patients are more likely to have offspring than severely affected patients. Another clear illustration of variable expression is Case 11, where two siblings with the same variant share a similar cognitive phenotype (moderate ID), whilst only one of them has congenital heart disease. Due to this variable phenotype, it can be challenging to distinguish truly benign variants from familial variants with high variable expression.

We show that DNA methylation studies and algorithms that model human facial dysmorphism and facial recognition can be helpful tools to determine the pathogenicity of such complex variants in several illustrative cases. Based on our experience, we propose an expansion of the evidence framework of the ACMG guidelines [6] (Box 1) to improve variant interpretation, which we discuss in more detail below.

Box 1 **Suggested updates of the ACMG guidelines for the interpretation of sequence variants.**

**PVS1.**

Adjusting the 2nd caveat of PVS1 into:

- Use caution interpreting LOF variants at the extreme 5′ and 3′ ends of a gene.

Adding **PS5**

A DNA methylation signature matching the gene in which the variant was identified is present.

Caveat:

- If variants in related genes lead to the same signature, care must be taken to exclude the relevant variants in those genes.

Adding **PM7**

Facial photograph clusters with photographs of patients with a confirmed pathogenic variant and separate from the matched controls.

### 4.2. Variant Location and the PVS1 Category

The PVS1 criterion in the ACMG framework is triggered when a predicted loss-of-function variant is detected in a gene known to have haploinsufficiency as a mechanism. One of the caveats to the PVS1 criterion is that 3′ variants may escape nonsense-mediated decay (NMD) [6,28]. Another problem is the possibility that 5′ loss-of-function variants may be rescued by an alternative translation start site. For *ARID1B*, we have previously noted that there are no convincing pathogenic variants reported at the 5′ end of exon 1 [7]. This could be a chance finding, could reflect that such variants are not pathogenic or perhaps that such variants are lethal and therefore not identified in live born patients. Since no BAFopathy DNA methylation signature was shown in Case 1, and the phenotypes of Cases 2 and 3 are not consistent with *ARID1B*-related ID, such a start site may be located between cDNA location 363 and 521. It may be that such a start site would rescue the complete phenotype, but an intriguing alternative is that it rescues part of the phenotype only. This could explain why we find a relative abundance of inherited variants in exon 1. There is one methionine in between (p.Met154), but other start codons have also been described to initiate translation [29]. We therefore propose to adjust the 2nd caveat of PVS1 to also include possibilities of alternative translation start sites: “Use caution interpreting LOF variants at the extreme 5′ and 3′ ends of a gene”.

The absence of pathogenic variants in exon 3 of *ARID1B* reinforces the importance of another caveat for PVS1: to use caution when the genes is known to generate multiple transcripts [6,30]. The in-frame exon 3 in transcript NM_020732.3 is not included in another *ARID1B* transcript NM_017519.2. This suggests that exon 3 is alternatively spliced and may not be required in all tissues, which is confirmed by several reported cases with loss-of-function variants in exon 3 without a matching phenotype [31,32].

Our Case 8, where in-frame exons 3 and 4 were deleted in an unaffected patient and parent, illustrates the importance of this caveat, but also of the caveat relating to skipping of in-frame exons. Interestingly, Pascolini et al. [33] describe monozygotic twins with an in-frame deletion of exon 2–4 of *ARID1B*, where the father could not be tested. The location, in-frame nature and unknown inheritance cast doubt upon the pathogenicity of this particular variant [34], although a very similar variant was identified in a CSS patient by Fujita et al. [35]. They identified an in-frame deletion of exons 2–5, of unknown inheritance, but the phenotype and the Face2Gene analysis (rank 1, similarity 0.62) make the pathogenicity of this variant more likely. Unfortunately, no DNA of this reported case could be obtained to determine the DNA methylation signature.

The described cases illustrate how important it is to keep in mind the caveats mentioned in the ACMG guidelines. For example, if these were to be disregarded, the variants of Cases 1, 2, 8 and 9 would have been classified as likely pathogenic or pathogenic instead of as a VUS. This also stresses the importance of having gene-specific knowledge concerning genotype and phenotype when interpreting variants.

### 4.3. Transcript Choice

We have considered switching to the MANE transcript for this paper. However, to prevent unnecessary confusion in the literature, we decided to only switch to a new transcript when it is fully backed up by clinical evidence (such as variants being identified in ‘new’ exonic regions (i.e. the additional 5’part of exon 1, and the in-frame exon 11). Thus far, this is not the case, although care should be taken that these ‘new’ regions are properly sequenced and called. Although they appear to be captured in most recent exome capture kits, it is possible that bio-informatic pipelines do not yet annotate these new regions causing clinical variants to be missed. We recommend that clinical labs check their pipelines for compatibility with the MANE transcript. We have also queried GnomAD for truncating variants in the new exonic regions, which would be an argument against the new transcript. We found five truncating variants in GnomAD (accession date 6 August 2021), but each variant was found once, and four of them are in low complexity regions, so this does not provide definitive evidence either way. Thus, even though NM_020732.3 is not the perfect transcript for *ARID1B* as it incorporates the in-frame exon 3, which is most likely not clinically relevant, we have chosen to keep using NM_020732.3 until there is more evidence for the new transcript. We have added the description of all variants detected in our cases on the MANE transcript in the Supplementary Data (Table S1). 

One important consequence of the new transcript is that the start codon variant of patient one changes into a missense variant (c.252G > A, p.(Met84Ile)). This might explain the absence of a DNA methylation signature in this patient, since for some genes specific signatures for missense variants are identified. The consequence of this variant is on the protein level is not yet known.

### 4.4. Incorporating New Developments into ACMG Guidelines for the Interpretation of Sequence Variants

Since the introduction of the ACMG guidelines there have been several relevant developments. Facial analysis has become more widely used and DNA methylation analysis has been shown to be an effective functional assay in clinical classification of an expanding number of genetic disorders [11,12], including CSS [10].

#### 4.4.1. Facial Analyses

By means of computer vision algorithms we have shown that facial photographs of *ARID1B* patients aged <10 years cluster separate from age-, sex-, ethnicity-matched controls with ID and that with advancing age this clustering becomes less pronounced (Figure S1). This apparent absence of a clear facial phenotype among older *ARID1B* patients could be explained by the notion that with advancing age the facial phenotype becomes less specific, but the low number of available photographs of older *ARID1B* patients may have played a role as well. We illustrated that facial analysis using childhood photographs can be helpful in the interpretation of difficult variants and we suggest that a positive facial gestalt match could be incorporated as a moderate ACMG criteria (PM7): “Facial photograph clusters with photographs of patients with confirmed pathogenic variants and separate from matched controls”. We think this better reflects the objective nature of facial analyses then allocating the PP4 criterion of a phenotype highly specific for gene. Of course, this criterion should be limited to the syndromes that display facial dysmorphism.

#### 4.4.2. DNA Methylation

Multiple studies have shown the potential of DNA methylation signatures for variant interpretation [11,12]. However, caution is warranted because the sensitivity of DNA methylation signatures may be not be complete for all pathogenic variants in a given gene. For example, multiple episignatures have been described in single genes in some disorders [36,37]. Similarly, the DNA methylation result of Case 1 is not compatible with a BAFopathy, whilst his photograph clusters with *ARID1B* patients and his phenotype may fit within the *ARID1B* spectrum. Hence, while existence of an episignature is generally considered strong functional evidence to confirm a pathogenicity of a variant, lack of an episignature, albeit indicative, should not be considered conclusive evidence of the lack of pathogenicity [12]. The DNA methylation results all mirror the assessment of pathogenicity before testing in this study, except for Case 4. In this case, a loss-of-function variant was found at the 5′ end of exon 1, at cDNA position 521. This variant was reclassified after the DNA methylation showed a BAFopathy methylation pattern in both the child and parent. These data, along with the growing body of published literature on other disorders, support incorporating DNA methylation as a strong criterion for classifying pathogenic variants (PS5): “A DNA methylation signature matching the gene in which the variant was identified is present”. In our view this should be a separate criterion from PS3 (well-established functional studies show a deleterious effect). This is also in line with ACGS Best Practice Guidelines for Variant Classification 2019 [38], since the evidence of a methylation signature supports phenotype specificity and is not directly related to the identified variant. Perhaps at a later stage when more is certain about the sensitivity of this DNA methylation test, a criterion could also be added to classify variants as benign in the absence of a DNA methylation signature.

#### 4.4.3. Incorporating Suggested ACMG Updates

Taking the suggested new PM7 and PS5 criteria and the adjusted PVS1 caveat into account, we have classified the variants and show that the pathogenicity assessment of the variants of our included cases would significantly change (Table 1) and have become more conforming to our interpretation of the *ARID1B* variants.

The UK Association for Clinical Genomic Science (UK-ACGS) publishes an annual ACMG-based specification for variant interpretation [39]. Using these criteria, the initial interpretation of the *ARID1B* variants would change for Cases 1, 2 and 4–7 into likely pathogenic and the interpretation using our updated ACMG criteria would remain unchanged.

## 5. Conclusions

Due to the variable phenotype among *ARID1B* patients, it can be challenging to distinguish familial variants with variable expression from benign variants. We urge caution in the interpretation of variants in the 5′ end of exon 1 and in-frame deletions and suggest to update the ACMG criteria PVS1 to also include caution with variants at the 5′ end of the transcript. We demonstrate the diagnostic utility of DNA methylation signatures in such cases and show the variable expression of the *ARID1B* phenotype, by reporting the first affected parent with normal IQ. Furthermore, we confirmed that *ARID1B* patients aged < 10 years have a distinct facial phenotype and found that the facial phenotype of *ARID1B* patients may become less specific with age. For this reason, if facial analyses are used to aid interpretation, childhood pictures should be preferred. Finally, we suggest the addition of facial analysis and/or DNA methylation signature analysis to the ACMG guidelines for interpretation of sequence variants in determining the impact of a genetic change on a patient’s diagnosis.

## Figures and Tables

**Table 1:** An overview of the included patients’ variants in *ARID1B* and a summary of the variant and phenotype assessments. (Transcript NM_020732.3).

Case	Exon	cDNA	Protein Change	Inheritance	Variant	GnomAD (1 May 2021)	DNA Methylation Pattern	Phenotype Suggestive for an *ARID1B* Related Disorder	Photograph Clusters with *ARID1B* Patients	CSS in Face2Gene (Rank/Similarity)	ACMG Criteria	Interpretation ACMG	Updated ACMG Criteria	Interpretation Updated ACMG	Expert Opinion
1	1	c.3G > A	p.(Met1?)	Not maternal	Start codon	No	no BAFopathy	+	+	2/0.32	PVS1, PM2	LP	PM2, PM7, PP4	VUS	VUS
2	1	c.361C > T	p.(Gln121*)	Paternal	Nonsense	No	n.a.	+/−	n.a.	n.a.	PVS1, PM2	LP	PM2, PM7	VUS	VUS
3	1	c.363_364insG	p.(Gln122fs*110)	Unknown	Frameshift	7x	n.a.	−	n.a.	n.a.	PVS1, BS2	VUS	PM4, BS2	VUS	LB
4	1	c.521dup	p.(Pro177fs*55)	Maternal	Frameshift	No	BAFopathy	+	+(parent−)	1/0.13 (−/0.13)	PVS1, PM2	LP	PS5, PM2, PM7	LP	P
5	1	c.1029_1056del	p.(Ala349Metfs*11)	Maternal	Frameshift	No	BAFopathy	+	+	19/0.08	PVS1, PM2	LP	PVS1, PS5, PM2, PM7	P	P
6	1	c.1044_1071del	p.(Ala349Metfs*11)	Maternal	Frameshift	No	n.a.	+	+	1/0.38	PVS1, PM2	LP	PVS1, PM2, PM7	P	P
7	1	c.1044_1062del	p.(Gly351Alafs*12)	Paternal	Frameshift	No	BAFopathy	+	+(parent−)	7/0.31 16/0.07	PVS1, PM2	LP	PVS1, PS5, PM2, PM7	P	P
8	3–4	exon 3–4 deletion	p.?	Paternal	In-frame deletion	-	no BAFopathy	-	n.a.	n.a.	PM2, PM4	VUS	PM2, PM4	VUS	LB
9	7	c.2371+2T > C	r.spl?	Unknown	Splice site	No	n.a.	+	n.a.	n.a.	PM2, PM4, PP4	VUS	PM2, PM4, PP4	VUS	VUS
10	8	c.2372-2A > C	r.spl?	Maternal	Splice site	1x	n.a.	+	n.a.	n.a.	PP4	VUS	PP4	VUS	VUS
11	18	c.4870C > T	p.(Arg1624*)	Father is inconclusively mosaic. Mother is negative. Siblings.	Nonsense	No	n.a.	+	n.a.	n.a.	PVS1, PM2, PP4	P	PVS1, PM2, PP4	P	P
12	20	c.6322C > T	p.(Gln2108*)	Paternal, mosaic father	Nonsense	No	n.a.	+	+(parent+)	1/0.78 1/0.34	PVS1, PM2, PP4	P	PVS1, PM2, PM7, PP1, PP4	P	P

Abbreviations: n.a.: not available; P: pathogenic; LP: likely pathogenic; VUS: variant of uncertain significance; LB: likely benign.

## Data Availability

Not applicable.

## Supplementary Materials

The following are available online at https://www.mdpi.com/article/10.3390/genes12081275/s1, Figure S1: (A) t-SNE plot depicting clustering of photographs of familial ARID1B cases (1 photograph per case was included) compared to confirmed ARID1B patients aged < 10 years photographs and age-, sex-, and ethnicity matched controls with intellectual disability. (B) t-SNE plot depicting clustering of photographs of familial ARID1B cases compared to confirmed ARID1B patients’ photographs and matched controls, Figure S2: depicting similarity and ranking of facial photographs of the familial *ARID1B* cases* in F2G. Table S1: overview of the clustering of photographs of *ARID1B* inherited cases in comparison to *ARID1B* outpatient clinic cases, Table S2: previously reported inherited loss-of-function *ARID1B* variants.

## References

[1] Hoyer J., Ekici A.B., Endele S., Popp B., Zweier C., Wiesener A., Wohlleber E., Dufke A., Rossier E., Petsch C. (2012). Haploinsufficiency of *ARID1B*, a member of the SWI/SNF-a chromatin-remodeling complex, is a frequent cause of intellectual disability. Am. J. Hum. Genet..

[2] Wright C.F., Fitzgerald T.W., Jones W.D., Clayton S., McRae J.F., van Kogelenberg M., King D.A., Ambridge K., Barrett D.M., Bayzetinova T. (2015). Genetic diagnosis of developmental disorders in the DDD study: A scalable analysis of genome-wide research data. Lancet.

[3] Santen G.W., Aten E., Vulto-van Silfhout A.T., Pottinger C., van Bon B.W., van Minderhout I.J., Snowdowne R., van der Lans C.A., Boogaard M., Linssen M.M. (2013). Coffin-Siris syndrome and the BAF complex: Genotype-phenotype study in 63 patients. Hum. Mutat..

[4] Wieczorek D., Bogershausen N., Beleggia F., Steiner-Haldenstatt S., Pohl E., Li Y., Milz E., Martin M., Thiele H., Altmuller J. (2013). A comprehensive molecular study on Coffin-Siris and Nicolaides-Baraitser syndromes identifies a broad molecular and clinical spectrum converging on altered chromatin remodeling. Hum. Mol. Genet..

[5] Tsurusaki Y., Okamoto N., Ohashi H., Mizuno S., Matsumoto N., Makita Y., Fukuda M., Isidor B., Perrier J., Aggarwal S. (2014). Coffin-Siris syndrome is a SWI/SNF complex disorder. Clin. Genet..

[6] Richards S., Aziz N., Bale S., Bick D., Das S., Gastier-Foster J., Grody W.W., Hegde M., Lyon E., Spector E. (2015). Standards and guidelines for the interpretation of sequence variants: A joint consensus recommendation of the American College of Medical Genetics and Genomics and the Association for Molecular Pathology. Genet. Med..

[7] Van der Sluijs P.J., Jansen S., Vergano S.A., Adachi-Fukuda M., Alanay Y., AlKindy A., Baban A., Bayat A., Beck-Wödl S., Berry K. (2018). The *ARID1B* spectrum in 143 patients: From nonsyndromic intellectual disability to Coffin–Siris syndrome. Genet. Med..

[8] Santen G.W., Clayton-Smith J., *ARID1B*-CSS Consortium (2014). The *ARID1B* phenotype: What we have learned so far. Am. J. Med. Genet. Part C Semin. Med. Genet..

[9] Van der Donk R., Jansen S., Schuurs-Hoeijmakers J.H.M., Koolen D.A., Goltstein L.C.M.J., Hoischen A., Brunner H.G., Kemmeren P., Nellåker C., Vissers L.E.L.M. (2019). Next-generation phenotyping using computer vision algorithms in rare genomic neurodevelopmental disorders. Genet. Med..

[10] Aref-Eshghi E., Bend E.G., Hood R.L., Schenkel L.C., Carere D.A., Chakrabarti R., Nagamani S.C.S., Cheung S.W., Campeau P.M., Prasad C. (2018). BAFopathies’ DNA methylation epi-signatures demonstrate diagnostic utility and functional continuum of Coffin-Siris and Nicolaides-Baraitser syndromes. Nat. Commun..

[11] Aref-Eshghi E., Kerkhof J., Pedro V.P., Groupe D.I.F., Barat-Houari M., Ruiz-Pallares N., Andrau J.C., Lacombe D., Van-Gils J., Fergelot P. (2020). Evaluation of DNA Methylation Episignatures for Diagnosis and Phenotype Correlations in 42 Mendelian Neurodevelopmental Disorders. Am. J. Hum. Genet..

[12] Sadikovic B., Levy M.A., Kerkhof J., Aref-Eshghi E., Schenkel L., Stuart A., McConkey H., Henneman P., Venema A., Schwartz C.E. (2021). Clinical epigenomics: Genome-wide DNA methylation analysis for the diagnosis of Mendelian disorders. Genet. Med..

[13] Levy M.A., Kerkhof J., Belmonte F.R., Kaufman B.A., Bhai P., Brady L., Bursztyn L., Tarnopolsky M., Rupar T., Sadikovic B. (2021). Validation and clinical performance of a combined nuclear-mitochondrial next-generation sequencing and copy number variant analysis panel in a Canadian population. Am. J. Med. Genet. Part A.

[14] Dingemans A.J.M., Stremmelaar D.E., van der Donk R., Vissers L., Koolen D.A., Rump P., Hehir-Kwa J.Y., de Vries B.B.A. (2021). Quantitative facial phenotyping for Koolen-de Vries and 22q11.2 deletion syndrome. Eur. J. Hum. Genet..

[15] Lord J., Gallone G., Short P.J., McRae J.F., Ironfield H., Wynn E.H., Gerety S.S., He L., Kerr B., Johnson D.S. (2019). Pathogenicity and selective constraint on variation near splice sites. Genome Res..

[16] Ruzzo E.K., Perez-Cano L., Jung J.Y., Wang L.K., Kashef-Haghighi D., Hartl C., Singh C., Xu J., Hoekstra J.N., Leventhal O. (2019). Inherited and *De novo* Genetic Risk for Autism Impacts Shared Networks. Cell.

[17] Stanley K.E., Giordano J., Thorsten V., Buchovecky C., Thomas A., Ganapathi M., Liao J., Dharmadhikari A.V., Revah-Politi A., Ernst M. (2020). Causal Genetic Variants in Stillbirth. N. Engl. J. Med..

[18] Al-Shamsi A., Hertecant J.L., Souid A.K., Al-Jasmi F.A. (2016). Whole exome sequencing diagnosis of inborn errors of metabolism and other disorders in United Arab Emirates. Orphanet J. Rare Dis..

[19] Stessman H.A., Xiong B., Coe B.P., Wang T., Hoekzema K., Fenckova M., Kvarnung M., Gerdts J., Trinh S., Cosemans N. (2017). Targeted sequencing identifies 91 neurodevelopmental-disorder risk genes with autism and developmental-disability biases. Nat. Genet..

[20] Sekiguchi F., Tsurusaki Y., Okamoto N., Teik K.W., Mizuno S., Suzumura H., Isidor B., Ong W.P., Haniffa M., White S.M. (2019). Genetic abnormalities in a large cohort of Coffin-Siris syndrome patients. J. Hum. Genet..

[21] Smith J.A., Holden K.R., Friez M.J., Jones J.R., Lyons M.J. (2016). A novel familial autosomal dominant mutation in *ARID1B* causing neurodevelopmental delays, short stature, and dysmorphic features. Am. J. Med. Genet. Part A.

[22] Mignot C., Moutard M.L., Rastetter A., Boutaud L., Heide S., Billette T., Doummar D., Garel C., Afenjar A., Jacquette A. (2016). *ARID1B* mutations are the major genetic cause of corpus callosum anomalies in patients with intellectual disability. Brain.

[23] Yu Y., Yao R., Wang L., Fan Y., Huang X., Hirschhorn J., Dauber A., Shen Y. (2015). *De novo* mutations in *ARID1B* associated with both syndromic and non-syndromic short stature. BMC Genom..

[24] Ben-Salem S., Sobreira N., Akawi N.A., Al-Shamsi A.M., John A., Pramathan T., Valle D., Ali B.R., Al-Gazali L. (2016). Gonadal mosaicism in *ARID1B* gene causes intellectual disability and dysmorphic features in three siblings. Am. J. Med. Genet. Part A.

[25] Cherot E., Keren B., Dubourg C., Carre W., Fradin M., Lavillaureix A., Afenjar A., Burglen L., Whalen S., Charles P. (2018). Using medical exome sequencing to identify the causes of neurodevelopmental disorders: Experience of 2 clinical units and 216 patients. Clin. Genet..

[26] Cheng S.S.W., Luk H.M., Mok M.T., Leung S.S., Lo I.F.M. (2021). Genotype and phenotype in 18 Chinese patients with Coffin-Siris syndrome. Am. J. Med. Genet. Part A.

[27] Min Z., Qian C., Ying D. (2021). Novel *ARID1B* variant inherited from somatogonadal mosaic mother in siblings with Coffin-Siris syndrome 1. Exp. Ther. Med..

[28] Abou Tayoun A.N., Pesaran T., DiStefano M.T., Oza A., Rehm H.L., Biesecker L.G., Harrison S.M., ClinGen Sequence Variant Interpretation Working G. (2018). Recommendations for interpreting the loss of function PVS1 ACMG/AMP variant criterion. Hum. Mutat..

[29] Kearse M.G., Wilusz J.E. (2017). Non-AUG translation: A new start for protein synthesis in eukaryotes. Genes Dev..

[30] Cummings B.B., Karczewski K.J., Kosmicki J.A., Seaby E.G., Watts N.A., Singer-Berk M., Mudge J.M., Karjalainen J., Satterstrom F.K., O’Donnell-Luria A.H. (2020). Transcript expression-aware annotation improves rare variant interpretation. Nature.

[31] Johnston J.J., Lewis K.L., Ng D., Singh L.N., Wynter J., Brewer C., Brooks B.P., Brownell I., Candotti F., Gonsalves S.G. (2015). Individualized iterative phenotyping for genome-wide analysis of loss-of-function mutations. Am. J. Hum. Genet..

[32] Yuen R.K.C., Merico D., Bookman M., Howe J.L., Thiruvahindrapuram B., Patel R.V., Whitney J., Deflaux N., Bingham J., Wang Z. (2017). Whole genome sequencing resource identifies 18 new candidate genes for autism spectrum disorder. Nat. Neurosci..

[33] Pascolini G., Valiante M., Bottillo I., Laino L., Fleischer N., Ferraris A., Grammatico P. (2019). Striking phenotypic overlap between Nicolaides-Baraitser and Coffin-Siris syndromes in monozygotic twins with *ARID1B* intragenic deletion. Eur. J. Med. Genet..

[34] Van der Sluijs P.J., Santen G.W.E. (2020). Letter regarding the article: Striking phenotypic overlap between Nicolaides-Baraitser and Coffin-Siris syndromes in monozygotic twins with *ARID1B* intragenic deletion. Eur. J. Med. Genet..

[35] Fujita T., Ihara Y., Hayashi H., Ishii A., Ideguchi H., Inoue T., Imaizumi T., Yamamoto T., Hirose S. (2020). Coffin-Siris syndrome with bilateral macular dysplasia caused by a novel exonic deletion in *ARID1B*. Congenit. Anom..

[36] Cappuccio G., Sayou C., Tanno P.L., Tisserant E., Bruel A.L., Kennani S.E., Sa J., Low K.J., Dias C., Havlovicova M. (2020). *De novo* SMARCA2 variants clustered outside the helicase domain cause a new recognizable syndrome with intellectual disability and blepharophimosis distinct from Nicolaides-Baraitser syndrome. Genet. Med..

[37] Bend E.G., Aref-Eshghi E., Everman D.B., Rogers R.C., Cathey S.S., Prijoles E.J., Lyons M.J., Davis H., Clarkson K., Gripp K.W. (2019). Gene domain-specific DNA methylation episignatures highlight distinct molecular entities of ADNP syndrome. Clin. Epigenetics.

[38] Ellard S., Baple E.L., Berry I., Forrester N., Turnbull C., Owens M., Eccles D.M., Abbs S., Scott R., Deans Z.C. ACGS Best Practice Guidelines for Variant Classification 2019: Association for Clinical Genetics Science (ACGS). https://www.acgs.uk.com/news/acgs-best-practice-guidelines-for-variant-classification-2019/.

[39] Ellard S., Baple E.L., Berry I., Forrester N., Turnbull C., Owens M., Eccles D.M., Abbs S., Scott R., Deans Z.C. ACGS Best Practice Guidelines for Variant Classification 2020: Association for Clinical Genetics Science (ACGS). https://www.acgs.uk.com/quality/best-practice-guidelines/#VariantGuidelines.

