# Supplementary material for: A Case Series of Familial ARID1B Variants Illustrating Variable Expression and Suggestions to Update the ACMG Criteria
Source: Genes (Basel). 2021 Aug 20;12(8):1275. doi: 10.3390/genes12081275 (PMC8393241; doi:10.3390/genes12081275)
Supplement: Supplementary file 1 [file genes-12-01275-s001.zip › 1genes-1288097_SUPPLEMENTARY-MATERIAL_18082021.pdf]

SUPPLEMENTARY MATERIAL

A Case Series of Familial *ARID1B* Variants Illustrating Variable Expression And Suggestions to Update the ACMG Criteria

PJ van der Sluijs, M Alders, AJM Dingemans, K Parbhoo, BW van Bon, JC Dempsey, D Doherty, JT den Dunnen, EH Gerkes, I Milller, S Moortgat, DS Regier, CAL Ruivenkamp, B Schmalz, T Smol, KE Stuurman, C Vincent-Delorme, BBA de Vries, B Sadikovic, SE Hickey, JA Rosenfeld, I Maystadt and GWE Santen

Contents

- Supplementary table S1 p. 3
- Supplementary table S2 p. 4
- Supplementary table S3 p. 6
- Supplementary figure S1 p. 7
- Supplementary figure S2 p. 9
- References p. 11

Supplementary table S1: overview of the variants of the *ARID1B* inherited cases on transcript NM_020732.3 and NM_001374828.1

Case	Genomic location (GRCh37)	cDNA change (NM_020732.3)	Protein change (NM_020732.3)	Inheritance	cDNA change (NM_001374828.1)	Protein change (NM_001374828.1)
1	g.157099066G>A	c.3G>A	p.(Met1?)	Not maternal	c.252G>A	p.(Met84Ile)
2	g.157099424C>T	c.361C>T	p.(Gln121*)	Paternal	c.610C>T	p.(Gln204*)
3	g.157099426_ 157099427insG	c.363_364insG	p.(Gln122fs*110)	Unknown	c.612_613insG	p.(Gln205Alafs*110)
4	g.157099584dup	c.521dup	p.(Pro177fs*55)	Maternal	c.770dup	p.(Pro260Alafs*55)
5	g.157100092_ 157100119del	c.1029_1056del	p.(Ala349Metfs*11)	Maternal	c.1278_1305del	p.(Ala432Metfs*11)
6	g.157100107_ 157100134del	c.1044_1071del	p.(Ala349Metfs*11)	Maternal	c.1293_1320del	p.(Ala432Metfs*11)
7	g.157100107_ 157100125del	c.1044_1062del	p.(Gly351Alafs*12)	Paternal	c.1293_1311del	p.(Gly434Alafs*12)
8	-	exon 3-4 deletion	p.?	Paternal	exon 3 deletion	p.?
9	g.157431697T>C	c.2371+2T>C	r.spl?	Unknown	c.2581+2T>C	r.spl?
10	g.157454160A>C	c.2372-2A>C*	r.spl?	Maternal	c.2582-2A>C	r.spl?
11	g.157522598C>T	c.4870C>T	p.(Arg1624*)	Father is inconclusively mosaic. Mother is negative. Siblings.	c.5239C>T	p.(Arg1747*)
12	g.157528597C>T	c.6322C>T	p.(Gln2108*)	Paternal, mosaic father	c.6691C>T	p.(Gln2231*)

Supplementary table S2: overview of the clustering of photographs of *ARID1B* inherited cases in comparison to *ARID1B* outpatient clinic cases

Case*	Photograph	Age (years)	Sex	*ARID1B* clustering (positive = like *ARID1B*, negative = like matched controls)	F2G rank	F2G similarity to CSS
1.1	1.1_1	20	male	positive	2	0.32
2.1	2.1_1	11.50	female	negative	7	0.29
	2.1_2	11.50		positive	8	0.31
	2.1_3	4.1		processing of photograph failed	1	0.58
	2.1_4	2.67		processing of photograph failed	2	0.13
	2.1_5	2.67		processing of photograph failed	5	0.13
	2.1_6	2.25		positive	2	0.14
2.2	2.2_1	47	male	processing of photograph failed	31	0.11
	2.2_2	47		negative	31	0.11
4.1	4.1_1	1.42	male	positive	1	0.13
4.2	4.2_1	36	female	negative	31	0.13
5.1	5.1_1	10	male	positive	19	0.08
6.1	6.1_1	4	female	positive	1	0.38
7.1	7.1_1	8	female	positive	1	0.20
	7.1_2	7		positive	7	0.31
	7.1_3	7		positive	1	0.31
	7.1_4	5		processing of photograph failed	1	0.59
	7.1_5	5		positive	1	0.83
	7.1_6	5		positive	1	0.54
	7.1_7	4		positive	2	0.17
	7.1_8	0.17		positive	1	0.56
	7.1_9	0.17		processing of photograph failed	1	0.24
	7.1_10	0.17		positive	1	0.43
7.2	7.2_1	25	male	negative	31	0.04
	7.2_2	14		negative	16	0.07
	7.2_3	8		processing of photograph failed	17	0.06
	7.2_4	7		negative	16	0.15
	7.2_5	3		processing of photograph failed	8	0.15
	7.2_6	1		negative	4	0.17

* [case number].1 is the proband/child, [case number].2 is the parent

Continuation of supplementary table S2: overview of the clustering of photographs of *ARID1B* inherited cases in comparison to *ARID1B* outpatient clinic cases

Case*	Photograph	Age (years)	Sex	*ARID1B* clustering (positive = like *ARID1B*, negative = like matched controls)	F2G rank	F2G similarity to CSS
12.1	12.1_1	8	male	positive	1	0.78
	12.1_2	3.58		positive	1	0.51
	12.1_3	3.17		positive	1	0.82
	12.1_4	2.25		positive	1	0.91
12.2	12.2_1	40	male	processing of photograph failed	7	0.10
	12.2_2	18		positive	1	0.34
	12.2_3	10		positive	1	0.54
	12.2_4	4		positive	2	0.23
	12.2_5	0.58		positive	1	0.37

* [case number].1 is the proband/child, [case number].2 is the parent

Supplementary table S3: previously reported inherited loss of function *ARID1B* variants.

Number of offspring carrying the variant	*ARID1B* variant	Exon	Mutation type	Patient's phenotype	Parent's phenotype	Reference	Pathogenicity
1	c.1259delA; p.(Asn420Ilefs*10)	1	Frameshift	CSS-like: ID, neurodevelopmental delays, short stature, and dysmorphic features	Similarly affected: neurodevelopmental delays, growth delay, and dysmorphic features	Smith 2016[1]	Pathogenic variant
1	c.1762G>T; p.(Glu588*)	3	Nonsense	n.r.	Unaffected	Johnston 2015[2]	Unlikely
2	c.1762G>T; p.(Glu588*)	3	Nonsense	Autism spectrum disorder, male monozygotic-twins	N.r./in mother	Yuen et al 2017[3]	n.r.
1	c.1927-2A>G	Intron 4	Splice site	Abnormal head shape; bone abnormality - osteopoikilosis; seasonal allergies; undescended testicle repaired at age 2; bipolar/mood disorder symptoms; OCD symptoms	N.r.	Yuen et al 2017[3]	n.r.
1	c.2037+46454_3551-1154del	6-13	Duplication	Short stature, language impairment, dysmorphic features, ID	N.r./in mother	Yu et al 2015[4]	Pathogenic variant
2	c.2038-52488_2371+613del	6-7	Deletion	CSS(-like), severe ID, coarse facial features, widely spaced teeth, neurodevelopmental delays, no speech, normal growth, seizures, hypertrichosis, hyperactivity	Unaffected; gonadal mosaicism proposed	Van der Sluijs 2018[5]	Pathogenic variant
2	c.2372-2A>C	Intron 7	Splice site	Autism spectrum disorder, no ID	N.r./in father	Ruzzo et al 2019[6]	n.r.
2	c.3468_3471del; p.(Glu1157Valfs*53)	13	Frameshift	ID, speech delay and dysmorphic features.	Unaffected/ mother, mosaic (4% of reads in blood)	Min et al 2021[7]	Pathogenic variant
2	c.4013+1G>A	Intron 16	Splice site	Autism spectrum disorder	N.r./in mother	Yuen et al 2017[3]	n.r.
3	c.4357C>T; p.(Gln1443*)	18	Nonsense	CSS-like: ID, dysmorphic features, 2 of the 3 sibling have hypoplasia corpus callosum	Unaffected; gonadal mosaicism proposed	Ben-Salem 2016[8]	Pathogenic variant
1	c.5830C>T; p.(Arg1944*)	20	Nonsense	Neurodevelopmental disorder	N.r./father mosaic (7% of reads in blood)	Cherot et al 2017[9]	Pathogenic variant
2	c.6726_6730del; p.(Leu2243Serfs*8)	20	Frameshift	CSS: mild to moderate ID, short stature, ASD, behaviour anomalies and dysmorphic features	Mildly affected/ mother mosaic	Cheng et al 2021[10]	Pathogenic variant

Used transcript: NM_020732.3

Abbreviations: CSS: Coffin-Siris syndrome, ID: intellectual disability, n.r.: not reported, OCD: obsessive compulsive disorder

Supplementary figure S1: (**A**) t-SNE plot depicting clustering of photographs of familial *ARID1B* cases (1 photograph per case was included) compared to confirmed *ARID1B* patients aged <10 years photographs and age-, sex-, and ethnicity matched controls with intellectual disability. (**B**) t-SNE plot depicting clustering of photographs of familial *ARID1B* cases compared to confirmed *ARID1B* patients' photographs and matched controls

t-SNE is a method for visualizing high-dimensional data. Since the hybrid model outputs a 468 dimensional feature vector, it is not possible to display these data on two axis, which only allow for the demonstration of two dimensions. t-SNE attempts to summarize the high-dimensional data in two dimensions, allowing visualization.

A

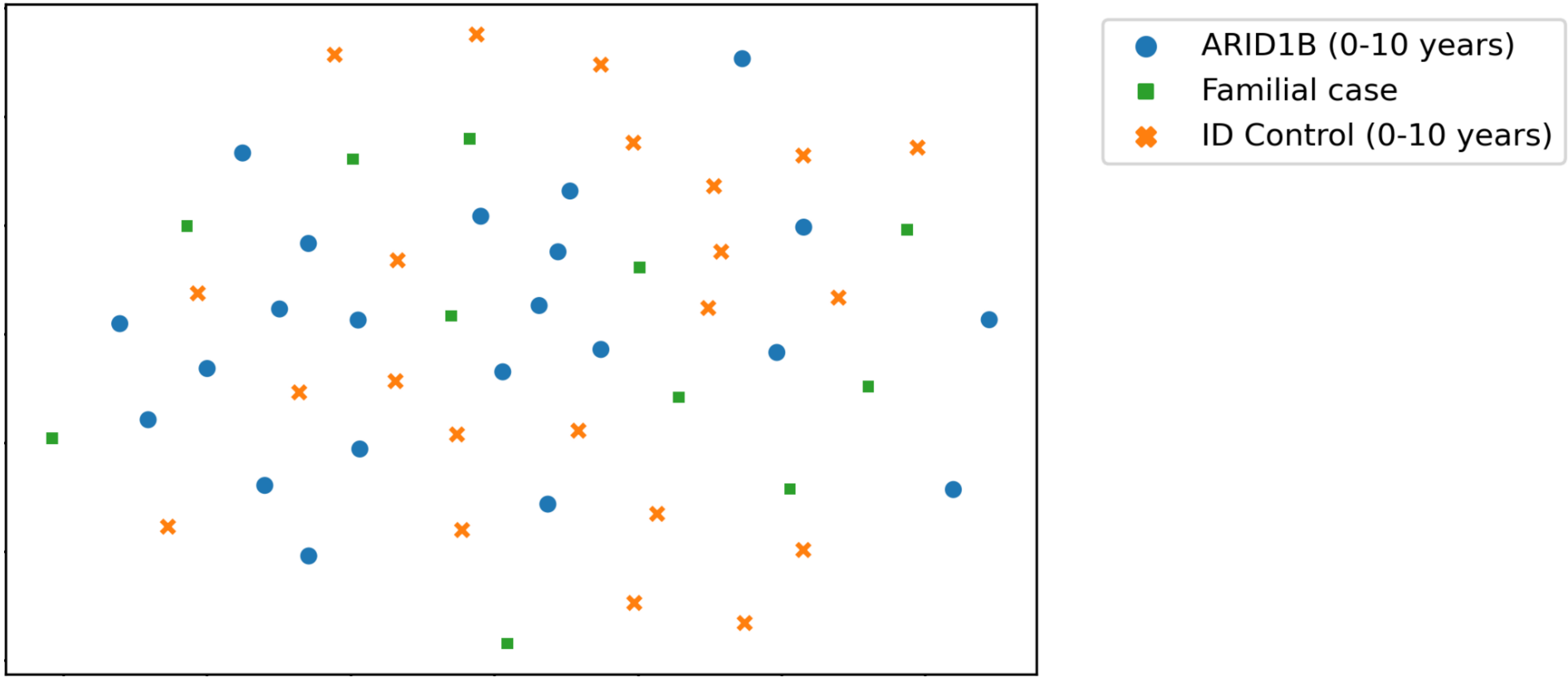

B

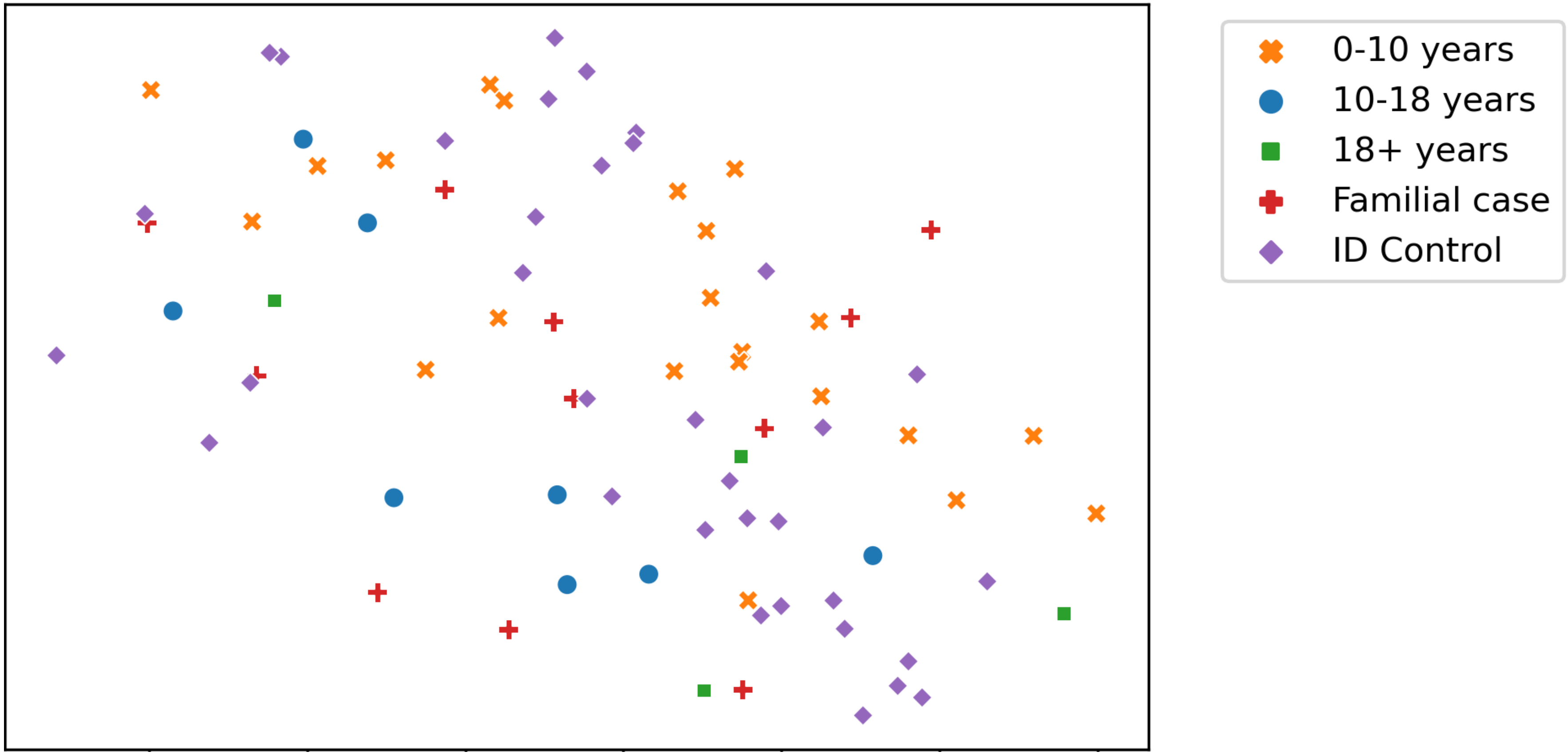

Supplementary figure S2: depicting similarity and ranking of facial photographs of the familial *ARID1B* cases* in F2G. (**A+B**)** Scatterplot depicting the similarity (**A**) or ranking (**B**) of facial photographs to CSS in F2G, one photograph per familial *ARID1B* case included. (**C + D**)** Scatterplot depicting the similarity (**C**) or ranking (**D**) of facial photographs to CSS in F2G of the familial *ARID1B* cases with multiple facial photographs available

A

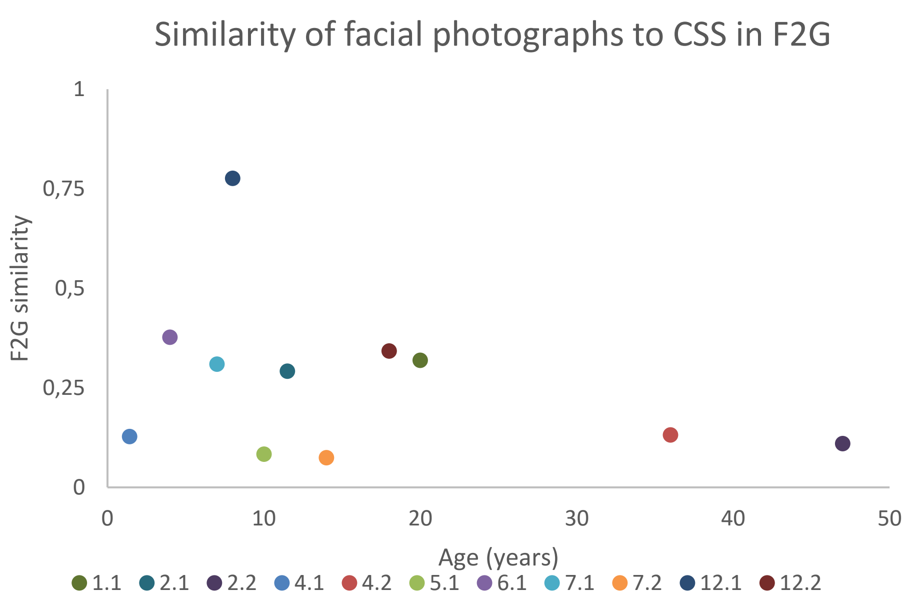

B

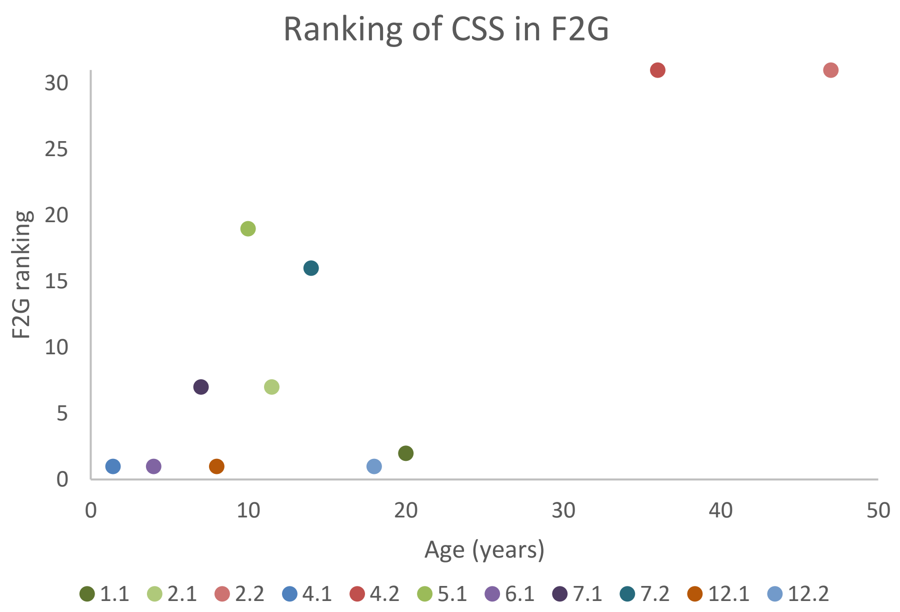

C

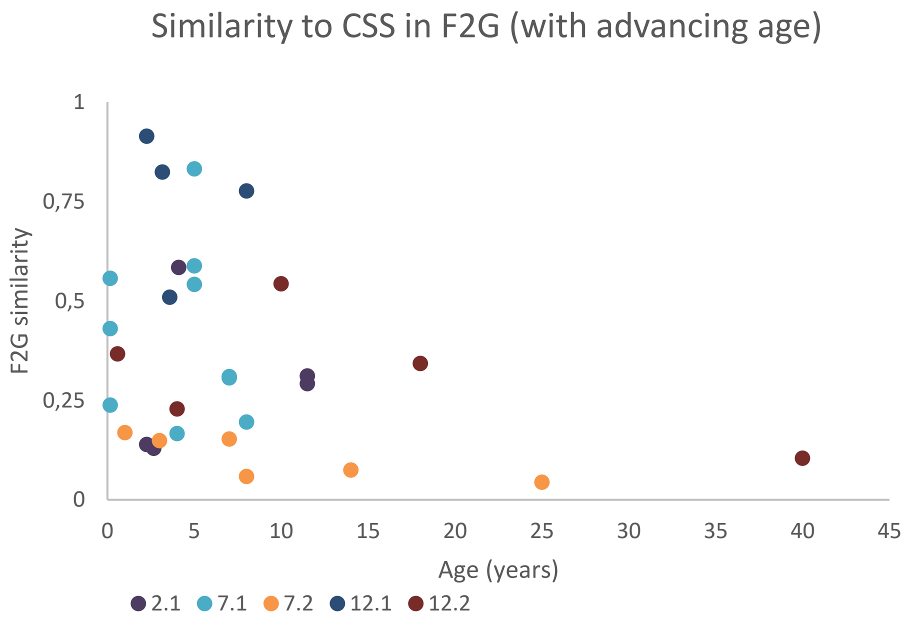

D

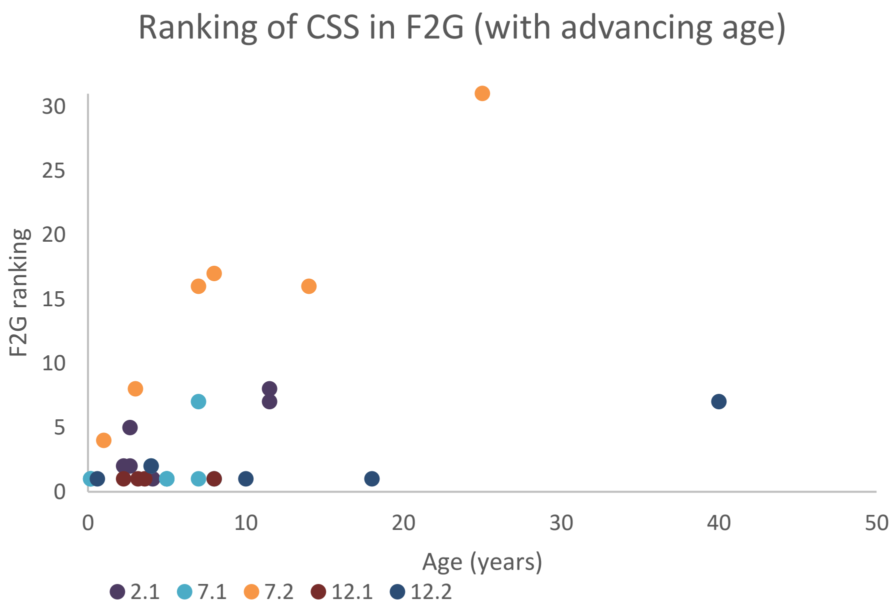

Abbreviations: CSS: Coffin-Siris syndrome, F2G: Face2Gene

* [casenumber].1 is child/proband (e.g. 7.1), [casenumber].2 is parent (e.g. 7.2)

** If Coffin-Siris syndrome was not in F2G's top 30 of suggested syndrome rank 31 was allocated in the figure

References:

1. Smith, J.A.; Holden, K.R.; Friez, M.J.; Jones, J.R.; Lyons, M.J. A novel familial autosomal dominant mutation in *ARID1B* causing neurodevelopmental delays, short stature, and dysmorphic features. *Am J Med Genet A* **2016**, *170*, 3313-3318, doi:10.1002/ajmg.a.37945.
2. Johnston, J.J.; Lewis, K.L.; Ng, D.; Singh, L.N.; Wynter, J.; Brewer, C.; Brooks, B.P.; Brownell, I.; Candotti, F.; Gonsalves, S.G.; et al. Individualized iterative phenotyping for genome-wide analysis of loss-of-function mutations. *Am J Hum Genet* **2015**, *96*, 913-925, doi:10.1016/j.ajhg.2015.04.013.
3. Yuen, R.K.C.; Merico, D.; Bookman, M.; J, L.H.; Thiruvahindrapuram, B.; Patel, R.V.; Whitney, J.; Deflaux, N.; Bingham, J.; Wang, Z.; et al. Whole genome sequencing resource identifies 18 new candidate genes for autism spectrum disorder. *Nat Neurosci* **2017**, *20*, 602-611, doi:10.1038/nn.4524.
4. Yu, Y.; Yao, R.; Wang, L.; Fan, Y.; Huang, X.; Hirschhorn, J.; Dauber, A.; Shen, Y. De novo mutations in *ARID1B* associated with both syndromic and non-syndromic short stature. *BMC Genomics* **2015**, *16*, 701, doi:10.1186/s12864-015-1898-1.
5. van der Sluijs, P.J.; Jansen, S.; Vergano, S.A.; Adachi-Fukuda, M.; Alanay, Y.; AlKindy, A.; Baban, A.; Bayat, A.; Beck-Wödl, S.; Berry, K.; et al. The *ARID1B* spectrum in 143 patients: from nonsyndromic intellectual disability to Coffin–Siris syndrome. *Genetics in Medicine* **2018**, doi:10.1038/s41436-018-0330-z.
6. Ruzzo, E.K.; Perez-Cano, L.; Jung, J.Y.; Wang, L.K.; Kashef-Haghighi, D.; Hartl, C.; Singh, C.; Xu, J.; Hoekstra, J.N.; Leventhal, O.; et al. Inherited and De Novo Genetic Risk for Autism Impacts Shared Networks. *Cell* **2019**, *178*, 850-866 e826, doi:10.1016/j.cell.2019.07.015.
7. Min, Z.; Qian, C.; Ying, D. Novel *ARID1B* variant inherited from somatogonadal mosaic mother in siblings with Coffin-Siris syndrome 1. *Exp Ther Med* **2021**, *21*, 614, doi:10.3892/etm.2021.10046.
8. Ben-Salem, S.; Sobreira, N.; Akawi, N.A.; Al-Shamsi, A.M.; John, A.; Pramathan, T.; Valle, D.; Ali, B.R.; Al-Gazali, L. Gonadal mosaicism in *ARID1B* gene causes intellectual disability and dysmorphic features in three siblings. *Am J Med Genet A* **2016**, *170A*, 156-161, doi:10.1002/ajmg.a.37405.
9. Cherot, E.; Keren, B.; Dubourg, C.; Carre, W.; Fradin, M.; Lavillaureix, A.; Afenjar, A.; Burglen, L.; Whalen, S.; Charles, P.; et al. Using medical exome sequencing to identify the causes of neurodevelopmental disorders: Experience of 2 clinical units and 216 patients. *Clin Genet* **2018**, *93*, 567-576, doi:10.1111/cge.13102.
10. Cheng, S.S.W.; Luk, H.M.; Mok, M.T.; Leung, S.S.; Lo, I.F.M. Genotype and phenotype in 18 Chinese patients with Coffin-Siris syndrome. *Am J Med Genet A* **2021**, doi:10.1002/ajmg.a.62187.
